# Exploring COVID‐19 experiences for persons with multiple sclerosis and carers: An Australian qualitative study

**DOI:** 10.1111/hex.13704

**Published:** 2023-01-13

**Authors:** Helen Correia, Pamela Martin‐Lynch, Marcia Finlayson, Yvonne C. Learmonth

**Affiliations:** ^1^ College of Science, Health, Engineering and Education Murdoch University Murdoch Western Australia Australia; ^2^ Psychological Sciences Australian College of Applied Professions Perth Western Australia Australia; ^3^ School of Rehabilitation Therapy Queen's University Kingston Ontario Canada; ^4^ Centre for Molecular Medicine and Innovative Therapeutics, and Centre for Healthy Aging, Health Futures Institute Murdoch University Murdoch Western Australia Australia; ^5^ Discipline of Exercise Science Murdoch University Murdoch Western Australia Australia; ^6^ Perron Institute for Neurological and Translational Science Nedlands Western Australia Australia

**Keywords:** carers, COVID‐19, multiple sclerosis, qualitative, resilience, stress

## Abstract

**Objective:**

The COVID‐19 pandemic continues to impact communities around the world. In this study, we explored the COVID‐19 experiences of persons with multiple sclerosis (MS) and carers.

**Methods:**

Using a qualitative approach, interviews were undertaken with 27 participants residing in Australia (10 persons with MS, 10 carers and 7 MS service providers). Demographic and background data were also collected. Interviews were analysed using an inductive iterative thematic analysis.

**Results:**

Across all groups, participants consistently recognized pandemic challenges and impacts for persons with MS and carers, especially due to disruption to routines and services. Emotional and mental health impacts were also highlighted, as anxiety, fear of contracting COVID‐19 and stress, including relationship stress between persons with MS and carers and family members. Some persons with MS also mentioned physical health impacts, while for carers, the challenge of disruptions included increased demands and reduced resources. In addition to acknowledging challenges, persons with MS and carers also gave examples of resilience. This included coping and adapting by finding new routines and creating space through rest and breaks and through appreciating positives including the benefits of access to telehealth.

**Conclusion:**

Additional support is required for persons with MS and carers in navigating the impacts of COVID‐19 as the pandemic progresses. In addition to addressing challenges and disruptions, such support should also acknowledge and support the resilience of people with MS and carers and enhance resilience through supporting strategies for coping and adaptation.

**Patient and Public Contribution:**

Service user stakeholders were consulted at the beginning and end of the study. They provided feedback on interview questions and participant engagement, as well as service user perspectives on the themes identified in the current study. Participants were provided with summaries of key themes identified and invited to provide comments.

## 1 INTRODUCTION

During the COVID‐19 pandemic, many communities have been dominated by public health directives to limit the spread of disease and reduce negative health impacts. In Australia, this included restrictions, such as lockdowns or limiting social mobility, as well as other directives such as mask‐wearing.^1^ The potential impact of COVID‐19 has been particularly salient for individuals who have existing chronic neurological conditions, such as multiple sclerosis (MS).^2^ In Australia, researchers estimated in 2017 that there were at least 25,000 persons with MS (103.7/100,000), increasing from previous estimates and varying across states, with the highest prevalence in Tasmania and the lowest in Queensland.^3^ Throughout the pandemic, public health directives meant that usual MS healthcare services were unavailable or substantially altered,^4^ with reduced outpatient and in‐home care services^5^ and increased reliance on telehealth.^4^ These service changes were received with varying acceptance across the MS community.^6^, ^7^

Research on the potential impacts for persons with MS during the pandemic have been mixed. Some report reductions in positive health behaviours, such as decreased physical activity, poorer sleep and dietary behaviours.^8^, ^9^ as well as increased levels of anxiety,^10^, ^11^, ^12^ depression^12^, ^13^, ^14^ and greater fatigue^14^ compared to persons without MS. Research also identifies COVID‐specific concerns such as fear of contracting the virus, perception of higher risk in persons with MS and increased loneliness associated with confinement and minimized social contact.^14^ Social concerns for persons with MS also extend to less social support, financial issues and employment concerns.^7^, ^13^

In contrast, a recent rapid systematic review argues that while anxiety, stress and depression may be higher in persons with MS during the pandemic compared to others, they may not be substantially different to prepandemic levels.^15^ Additional research shows minimal MS symptom change,^7^ as well as reporting positive impacts of COVID‐19, including increased family time and improved capacity to manage fatigue.^7^, ^16^ Some research also recognizes individual and social contexts that support coping and resilience during the pandemic.^17^ Persons with MS may employ supportive strategies such as active coping and acceptance^17^ and maintain health behaviours such as physical exercise that reduce the impact of disability and depression in this context.^8^, ^18^ Together, these behaviours may result in greater resilience, predicting less depression and anxiety.^10^

Family and care of persons with MS may also be impacted during a pandemic. Restrictions may dramatically change daily interpersonal environments and disruptions to support services are often addressed by the immediate family. Given the importance of the relational network of persons with MS,^19^ an understanding of this social context is imperative, yet research is limited. Research looking at informal carers of people with various chronic health conditions in the context of the COVID‐19 pandemic reported an increase in caregiving intensity and burden^20^ as well as greater symptoms of depression, anxiety, fatigue and sleep problems compared to noncarers.^21^ For MS carers specifically, research recognizes increased responsibility for care in response to limited home services, coupled with the lack of support for these family members to care for their own health and well‐being.^7^ Australian and European caregivers of persons with MS express increased anxiety about worsening MS symptoms in their loved ones and fears of COVID‐19.^7^, ^16^ To date, there appear to be no other studies that focus on pandemic experiences and impacts for carers or family members of persons with MS.

There are concerns more generally about declines in carer psychological well‐being in the absence of sufficient support for family carers during the pandemic,^22^ with international calls for carer support and caregiver‐centred interventions.^22^, ^23^ Yet there is little research specifically on the impact of COVID‐19 on MS carer and family experiences to guide the development of such interventions tailored for the MS community. Using a qualitative approach to deepen understanding of the issues, this study builds on previous research by integrating multiple perspectives: persons with MS, carers and family members and MS service providers. MS service providers can provide a key perspective on potential impacts such as service access and engagement, especially in the context of responding to community crises such as COVID‐19.^7^ To date, there appear to be no other studies that integrate the service provider perspective in relation to carer experiences during COVID‐19. The primary aim of the current study was to explore how the pandemic influenced the experiences of persons with MS and MS carers and loved ones, including changes to the care experience in both personal and service contexts.

## 2 METHODS

### 2.1 Study design

This study was part of a broader qualitative study investigating caregiving experiences in MS. For the current study, the data set focused on a subsection of the data corpus in which participants commented specifically on their experience during the pandemic and how the pandemic had impacted care experiences. A university ethics review board granted approval for the study, and consumer representatives provided input into the study design and interview process. All participants received relevant study information and provided consent before participating in interviews. Participation in the interviews was voluntary, without remuneration. Interviews were conducted between September 2020 and August 2021. During this time, the different states and territories of Australia were experiencing varying levels of pandemic impacts, from low levels of transmission, restriction and minimal social disruption (e.g., Western Australia) to higher levels of transmission and government‐directed societal lockdown (e.g., Victoria).

### 2.2 Participant recruitment

**Figure 1:**
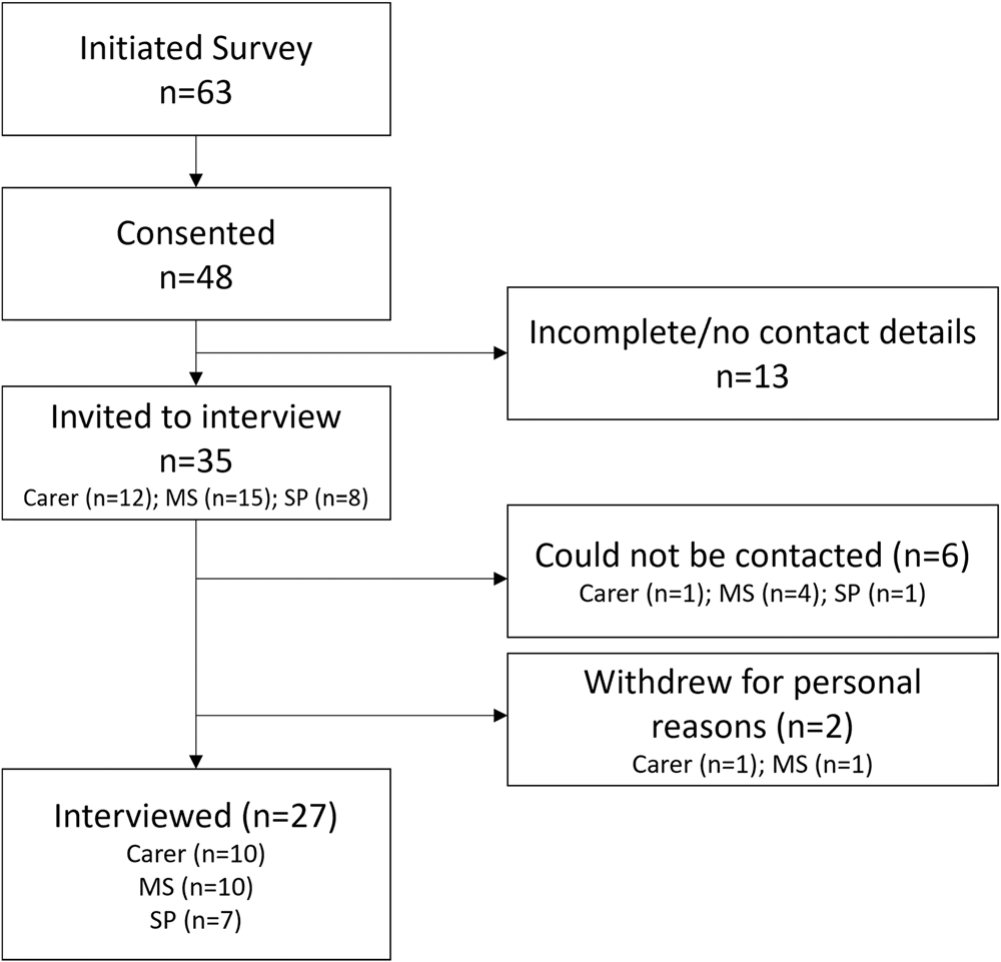
Participation throughout the study (MS: person with Multiple Sclerosis; SP: service provider).

To sample from a broad range of perspectives, we recruited participants across Australia from three different groups: persons with MS, carers/loved ones and services (e.g., health professionals, support service providers). Potential participants with MS and carer participants were recruited through electronic advertising (e.g., newsletters, social media) via MS organizations, as well as a database of participants in previous studies who had consented to be contacted about future studies. Potential participants from services were also recruited via MS Australia and the Australian state‐based MS organization (i.e., via social media announcements and emails). All interested individuals were directed to an online survey site (Qualtrics), which provided information about the study and collected basic information (demographics, background information) as well as consent to being contacted for the interview. A flow chart of participation through to interview can be seen in Figure 1.

Persons with MS could be included if they were above 18 years of age, had an established definite diagnosis of MS and received regular support or assistance with activities from carers (e.g., transportation, shopping and meal preparation). Carers could be included if they were above 18 years of age, had a close connection to a person with an established diagnosis of MS and had regular and frequent contact to provide care and support for the person with MS. Service providers could be included if they were involved in health‐related services/management of persons with MS (including health professionals, service managers or MS community support and advocacy). As individuals who have ongoing contact with persons with MS and their carers, this group provides an additional perspective on experiences such as changes to engagement in services during the pandemic. To capture the broadest range of experiences, individuals were not required to be linked to each other.

### 2.3 Participants

Thirty‐five individuals provided contact information for the interview (15 persons with MS; 12 Carers; 8 service providers), but six could not be contacted. One person with MS and one carer withdrew for personal reasons before the interview could be conducted. Twenty‐seven participants completed interviews (10 persons with MS; 10 carers; 7 service providers). To our knowledge, the participants were independent of each other. This sample represented all the individuals who expressed an interest and could be contacted before the study closing.

Participants identified being in age groups from 25 to 75 years of age. Participants with MS and carer participants predominantly identified their partner/spouse as the main caregiving relationship, except for one person with MS who identified their friend as the main carer and two carers identifying their adult child with MS as the person they support. Participants with MS were represented across age groups 25–75 years of age, while carers were mostly older (55–75 years of age) and service providers were mostly younger (25–44 years of age). Participants across all three groups were predominantly female (85% of all participants).

Participants were recruited from across Australia and from most states and territories. Most service providers were healthcare practitioners (four physiotherapists, one counsellor, two coordinators of well‐being/disability support for persons with MS and carers). All service providers reported seeing clients with a variety of MS types and disability levels. See Table 1 for participant characteristics across groups.

**Table 1:** Participant information

	MS	Carer/family	Service provider
Participants	10	10	7
Gender			
Female	9	8	6
Male	1	2	1
Age			
25–34	2	1	3
35–44	3	1	3
45–54	2	1	0
55–64	2	5	1
65–74	1	2	0
State/territory^a^			
Australian Capital Territory	2	0	0
New South Wales	2	4	0
Queensland	1	1	1
South Australia	1	0	0
Victoria	1	2	2
Western Australia	3	3	4
MS type of person with MS^b^			
Relapse remitting	8	5	‐
Primary progressive	1	1	‐
Secondary progressive	1	4	‐
Years since the diagnosis of person with MS^b^			
0–4	2	2	‐
5–9	3	3	‐
10–14	3	0	‐
15–19	1	3	‐
20–24	1	0	‐
25–30	0	1	‐
30 or more	0	1	‐
Disability (PDDS) of person with MS^b^			
Mild	3	2	‐
Moderate	6	2	‐
Severe	1	6	‐
Carer‐burden (CTiMMS means)^c^			
Activities of daily living	‐	1.83	‐
Instrumental care	‐	3.20	‐
Psycho‐emotional care	‐	2.48	‐
Social‐practical care	‐	2.53	‐

Abbreviations: MS, multiple sclerosis; PDDS, Patient Determined Disease Steps.

^a^ No participants were from Tasmania or Northern Territory.

^b^ MS‐related information from carers refers to the person with MS that they provide care for.

^c^ CTiMMS range: 0 (No help)–4 (lots of help).

### 2.4 Procedure

Interviews were conducted by two members of the research team (H. C. and P. M.‐L.), who have experience in interviewing and qualitative research. None of the participants had a pre‐established relationship with the interviewers. Question development was informed by previous research, reviewed following initial pilot interviews and feedback from stakeholder representatives (a person with MS and a carer). As part of the broader semistructured interview, participants were asked questions relating to caregiving and carer involvement in health care for persons with MS. Interviews were conducted by phone and lasted between 30 and 50 min (average of 42 min). For the current study, participants were invited to comment on the impact of COVID‐19 personally and any changes to the care experience that they had observed. The invitation was an open‐ended discussion and general, allowing for variability in impact given the diversity of geographical experiences with the pandemic (e.g., exposure to the virus, health directives, lockdown duration, restrictions). Clarifying questions were based on participants' responses. All interviews were audio‐recorded and transcribed for analysis, removing any identifying information. Following each interview, the researcher created a summary of key ideas identified in the interview and sent it to the participant with an invitation to review and provide feedback or to provide further comments. Any returned comments were incorporated into transcripts for analysis.

### 2.5 Demographic and clinical descriptors

During initial recruitment via our online recruitment survey, we gathered data on gender, age and state or territory from all participants. From persons with MS, we gathered data on MS type at diagnosis, years since diagnosis, disability level and carer context (relationship type, living arrangements, etc). The level of disability was gathered using the Patient Determined Disease Steps (PDDS), which correlates highly (*r* = .78) with the clinician‐rated Expanded Disability Status Scale.^24^ We categorized these into no or mild disability (score of 0–2), moderate disability (score of 3–5) and severe disability (score of 6–8). For carers, we asked about the number of years they had been a carer for their associated persons with MS, what type of MS the person has and the level of carer support. Levels of carer support were established using the Caregiving Tasks in MS Scale (CTiMSS).^25^ The CTiMSS comprises 24 items measuring the amount of help provided to care recipients on scales from no help (0) to lots of help (4). These are grouped into four domains assessing basic activities of daily living (e.g., toileting, feeding), instrumental activities of daily living care (e.g., preparing meals, transportation), psycho‐emotional care (e.g., managing fatigue, mood swings) and social‐practical care (e.g., providing companionship, assisting with physical exercises). Mean scores were calculated for each subscale, with higher ratings indicating more carers' help in given tasks. Service providers were asked to describe their work role and their experience working with persons with MS.

### 2.6 Data analysis

The current study analysed the data set from interviews specifically pertaining to COVID‐19 experiences. This was predominantly generated in response to the specific question on COVID‐19 at the end of the interview. To maximize the data set, spontaneous comments relating to COVID‐19 experiences provided in other parts of the interview were also included. Analysis of transcripts was undertaken using NVivo software (Release 1, 2021; QSR International Pty. Ltd.). Given the exploratory nature of the research, coding and development of themes followed an inductive iterative process used in thematic analysis^26^ with steps to enhance trustworthiness and rigour.^27^ Analysis was positioned as experiential and inductive and incorporated both semantic and latent coding.^28^ The stages of analysis included initial analysis and development of preliminary codes undertaken by two members of the research team (H. C. and P. M.‐L.); review of open coding and development of a broad framework; review of emerging themes and subthemes with reference to the raw data; producing the report. To support triangulation across these stages, code and theme development was discussed between coders, with further review and input through regular meetings with members of the research team. Findings have been reported using COREQ guidelines for qualitative research.^29^

## 3 RESULTS

Several key themes were identified in the data including acknowledging *challenges and impacts*, *coping and adapting* to the challenges of COVID‐19 and *appreciating positives* despite the challenges that emerged because of the pandemic. Table 2 provides a summary of key themes, subthemes and examples of codes.

**Table 2:** Overall themes, subthemes and example codes across perspectives: Participants with MS, carers/family, MS service providers

Themes	Subthemes	Example codes
Challenges and impacts	Disruptions	Personal disruptions; social disruptions; service disruptions
	Physical and mental health	Fear of infections; anxiety and stress; mental health challenges; physical health impacts
	Reduced resources	Demands and pressures; increased carer burden; lack of support; energy and fatigue
	Relational and social	Isolation; living pressures; relational stress; employment impact
Coping and adapting	Creating space	Getting out; respite and taking breaks; time apart
	Routines and activities	Maintaining and replacing activities; adapting to telehealth; maintaining physical health; self‐care; social activities
Appreciating positives	Service continuity	Maintaining access to services
	Technology supports	Telehealth; online connections
	Strengthening relationships	Time together; becoming closer

Abbreviation: MS, multiple sclerosis.

### 3.1 Consistency of themes across groups

Each major theme was reflected in responses from participants from all three groups and was present in responses across more than half of those interviewed. Interestingly, service providers' comments predominantly focused on challenges and impacts, especially subthemes relating to pandemic‐related disruptions and impacts on emotional and mental health. Ideas relating to the main themes of coping and adapting and appreciating positives were more often raised by persons with MS and carers and family members rather than service providers. Group variations in themes and coding are expanded in the sections below.

### 3.2 Challenges and impacts

The strongest theme described pandemic‐related challenges and impacts. This included disruptions to services and personal routines due to health directives such as lockdowns and restrictions. Some participants also described behavioural changes due to personal fears and concerns about COVID‐19.

#### 3.2.1 Disruptions

Participants across all groups typically highlighted observed disruptions as a result of COVID‐19, especially service disruption for persons with MS. Some participants highlighted the impact on physical health and social engagement. We also heard that while telehealth was welcomed, it was not always easy or sufficient.

But I think what one of the major things that has a negative effect on (husband with MS) is, say, neuro physio and things like that, that's all been kind of cancelled, which has happened before in the past, which does have quite an impact on his body, quite a bit. (Carer, Female)

The disruptions in personal routines and social events for persons with MS as well as carers and family members were also noted. This included personal health routines, travel, recreational activities and family events. In some cases, access to these activities appeared to be due to limits imposed through health directives, and in other cases, it was connected to concerns about COVID‐19 itself.

Well before Covid I was actually doing an exercise program with MS but because of possibly picking up a bug in a gym I basically stopped going back. So I suppose that that was the biggest impact. When it started I haven't been bothered to go back. (MS, Male, severe disability)

I think the main one is probably the socialisation aspect. There's a lot of people too scared to go out. There's a lot of care support workers who don't do social things with clients now because of all the fears of the bugs being transmitted, the virus being transmitted around. So if anything, we've lost a lot of socialisation. (Service Provider, Female)

As suggested in the excerpts, we also heard from participants about the personal and social impact of these challenges on their physical, mental and relational health.

#### 3.2.2 Physical and mental health

Participants across all groups commented on the emotional response to the pandemic and its challenges, as well as concerns about the physical and mental health of persons with MS and carers/family members. These connected both directly to the pandemic (e.g., fears of infection), as described previously, because of responses to the pandemic, such as disruption to services or reduced access to activities. The interviews included reference to a broad range of emotions such as fear, anxiety, stress, frustration and depression, including personal experiences in themselves or observed in persons with MS and carers and family members.

So, it would be nice to have the fear and concern not as kind of like an everyday thought and concern. (Carer, Female)

We moved treatment dates around because we didn't want to be too immuno‐compromised or coming into the hospital during Covid outbreaks, and so that is stressful, like I think I'm more stressed and anxious than I would have been otherwise and so that obviously then has an impact because it just means everything is harder to navigate, blood tests are harder to navigate, so it does make me grumpier I think. (MS, Female, moderate disability)

Participants described health concerns about contracting COVID‐19, describing their perceptions of higher risk and how this may influence their choices in accessing services and engaging in activities.

#### 3.2.3 Reduced resources

Participants across all groups acknowledged that disruption to MS‐related services was often accompanied by increased demands and lack of support, especially for immediate family and carers who assumed the support that would ordinarily be provided by services. We noted observations of the emotional and physical impact of navigating the pandemic, placing strain on personal resources.

It's a lot harder obviously being at home and with the treatment I wasn't very mobile either so it was so much heavier on him (participant's carer)—he had to do everything really for at least 3 months. (MS, Female, moderate disability)

A feeling of, you know that carer's burnout are really coming to fruition where the supports weren't able to come into the home and they had to do everything themselves and the quality of their health declining as well and their ability to look after themselves, declining. So, you know, onset of depression, symptoms of anxiety going, ‘How am I going to do this on my own?’ was a big one, especially if the individual with MS also had mental health issues. (Service Provider, Female)

Importantly, in the context of COVID‐19, carer participants rarely described this as an additional burden, instead, there was more recognition that this was a necessary change, whilst acknowledging it was a difficult challenge.

#### 3.2.4 Social and relational impacts

In some interviews, participants reported impacts on their own relationships as pandemic‐related constraints, such as lockdowns, increased living pressures and relationship stress. For other participants, the experience of restrictions was linked to isolation and loneliness, particularly for persons with MS.

So, my kids were off still doing their days because if they were working or whatever, but for me it was total isolation. So, it was very depressing. And your self‐worth goes into your boots, I was lonely. (MS, Female, moderate disability)

And probably I'm seeing a family, you know, people are working from home, the carers are working from home because people can't go out as much possibly for all families, but especially ones with disability, and MS that it puts more strain on the relationship from just living in each other's pockets. (Service Provider, Female)

Participants in our interviews described a broad range of experiences, challenges and difficulties in response to the pandemic, some related to pandemic‐related restrictions and the flow on effects of these and others related to concerns about contracting COVID‐19 directly. While these concerns and impacts understandably dominated the descriptions of the COVID‐19 experience, they were not solely defined by complaint or helplessness, rather we also heard in participant interview responses intended to promote coping and adaptation to very difficult circumstances.

### 3.3 Coping and adapting

Across interviews with persons with MS and carers, we heard examples of attempting to respond to the pandemic and related restrictions in helpful ways, such as by creating space and through routines and activities.

#### 3.3.1 Creating space

In the idea of creating space through the pandemic, participants referred to the impact of lockdowns and trying to manage confinement, highlighting the importance of space, either in physically different locations or in finding space for self. We heard descriptions from participants about the importance of ‘getting out’, and for carers, the need for setting boundaries, finding respite, taking breaks, spending time apart and having space and time for themselves.

It's a little concerning, like all these issues. High risk for COVID. And, um, yeah, it's putting a bit more stress on us to be at home, like locked down 24/7 together. Um, so we've kind of been taking time apart, you know, for her to watch her TV shows and for me to play video games like we kind of separate into our own thing. (Carer, Female)

So consequently, she's sort of locked herself in since then and we haven't been out. And I get out by going shopping and I mean, shopping for food and that sort of thing. I don't buy big lots, so I go down three or four times a week. (Carer, Male)

#### 3.3.2 Routines and activities

The above excerpts also highlight for some participants the importance of maintaining meaningful activities and finding alternative ways of meeting physical health and social needs. We heard in some interviews of persons with MS and carers the importance of maintaining routines and activities to meet these needs.

I would go, drive to the coffee shop, take my walking pole and get a takeaway coffee and go for a walk for half an hour, so I developed a routine in my day and that routine was what basically saved my mental health. Otherwise, I wouldn't have seen anyone. So that coffee man became so important. (MS, Female, moderate disability)

Overall, we heard from some participants examples of efforts to overcome initial challenges, disruptions or personal concerns, to navigate the pandemic in helpful and adaptive ways. Similarly, in some interviews, we also noted participants' efforts to recognize and appreciate positive experiences and unexpected benefits.

### 3.4 Appreciating positive experiences and benefits

In interviews with MS and carers and family members, and occasionally service providers, there was some recognition of positive experiences. This included, for example, experiences that offset challenges, such as service continuity rather than service disruption. A few participants also observed strengthening of relationships in the pandemic context. For others, benefits emerged in response to the pandemic, such as the use of technology for telehealth or to maintain social connection, as well as to help manage time and effort associated with face‐to‐face events.

Our access to caregiving has been great, sometimes they're all maxed up and everything, but they have been able to come. But auxiliary services sometimes didn't come like the house cleaning and things but that wasn't really an issue. (Carer, Female)

The fact that he can make an appointment with our GP and instead of having to use up all his energy available that day on just getting to the GP to have a meeting for just a script and that's it. The fact that he could have a five‐minute conversation and it's done is just brilliant. A lot of the museums and the zoo's and things like that were online, and he was able to do that with the kids which was fantastic. (Carer, Female)

In interviews, we noted that there was an acknowledgement of positive experiences, and we also heard positive evaluations in appreciation and gratitude, through descriptive words such as *great, lucky, bonus, thankfully and brilliant*. Examples of coping and resilience were minimally present in service providers' observation, who tended to focus predominantly on the experiences of challenges and negative impacts on persons with MS and carers.

## 4 DISCUSSION

The current study brought together perspectives of persons with MS, carers and family members and service providers, to explore pandemic‐related experiences of persons with MS and carers and family members. With limited research on MS carers during the COVID‐19 pandemic,^7^, ^16^ this study used qualitative methods to gain a richer understanding of the MS carer experience during this time. We heard salient themes surrounding the challenges of disruptions, as well as impacts on health, relationships and resources. Yet we also heard examples of resilience, with persons with MS and carers finding ways to cope and adapt and appreciating positive experiences and benefits gained amidst the pandemic. Interestingly reports of positive adaptation tended to be absent from accounts of service providers, which may reflect a potential blind spot in their knowledge of the pandemic experience for persons with MS and their carers.

Many key themes identified here are reflected in previous studies, including disruptions due to COVID and the significant shift away from home services towards telehealth.^4^, ^5^, ^30^ As with our research, MS participants in previous studies welcomed telehealth as a method of increasing access to services,^6^ although concerns remain regarding ease of use for some people recognizing that it is not always an adequate replacement.^7^ As the world adapts to the current pandemic and how it may unfold, further research is needed that gains a more nuanced understanding of telehealth use for the MS community, to ensure equitable development in response to diversity in ability, economic resources and metropolitan proximity.

In describing salient impacts of the pandemic, in the current study, the emotional, physical and mental health impacts for persons with MS partly echo previous research identifying experiences such as anxiety and stress^12^, ^13^, ^14^ and COVID‐related fear and concerns.^10^, ^11^, ^12^ Unlike some studies,^14^ there was not a strong theme of fatigue for persons with MS. This may be partly due to participant self‐selection, where individuals with significantly low mood and fatigue are perhaps less likely to engage in interviews. However, there were not consistently strong reports of low mood, fatigue or sleep problems from carer and service provider participants about persons with MS. Rather, anxiety and stress were a more common theme for MS participants, but further research is needed to monitor changes as communities progress through different phases of the pandemic.

This study adds to the existing literature by providing greater insight into COVID‐19 pandemic experiences for MS carers and families. Their experiences of additional demands due to service disruptions were highlighted by persons with MS, service providers and carers themselves. This is consistent with the experiences of carers with other conditions during COVID‐19^20^ as well as MS carers in previous studies.^7^ Similar to impacts on persons with MS, carers and family members acknowledged stress and anxiety, as reported in previous research,^16^ although unlike previous research,^7^, ^22^ carers and family members in the current study did not specifically report feeling less supported as a result of the pandemic. Nevertheless, reports of increased demands with reduced resources for MS carers add weight to the need for carer‐centred interventions^22^, ^23^ that support resourcing and resilience. There is also a benefit in exploring the role of broader MS relational networks^19^ and their experience in these times.

Previous research has identified factors that may shape different experiences of the pandemic, such as perceived COVID‐19 risk and personal resilience,^10^ coping and acceptance^17^ and recognizing benefits as well as challenges of pandemic‐related restrictions.^7^, ^16^ The current study provides some insight into possible facilitating strategies, such as creating physical or emotional space, developing routines and activities or through appreciation and gratitude. Some of these strategies have historically demonstrated potential benefits in other contexts.^31^, ^32^ This suggests a need to bolster MS community research and services that explore coping, adaptation, resilience and positive resourcing at multiple levels, including personally, relationally and systemically.

There are important limitations. First, interviewees predominantly indicated they were female, which may influence reported experiences and responses, including in coping and adaptation. Future studies should consider gender differences, as well as the impact of culture on care expectations and perceptions. In addition, while experiences were sampled across the country, adding to the diversity and richness of different experiences, it also impacts the consistency of findings. COVID‐19 impacts such as service delivery, social isolation and disconnection from usual activities were reported more by participants experiencing significant lockdowns and restrictions, yet these experiences were subject to geographical and temporal variability as the pandemic progressed and health directives changed across the course of the study. Similarly, there were differences between groups in experiential variables (e.g., age, length of diagnosis, location), which may have introduced additional variability. This, along with the small sample size, limited any kind of subgroup analyses. As the pandemic evolves, future research may need to consider a more refined process for evaluating the impact of pandemic‐related variables on persons with MS and their carers and family members, especially as we prepare for potential future pandemics.

## 5 CONCLUSION

Overall, the current study is relatively unique in providing a focused qualitative exploration of COVID‐19 impacts and experiences of MS carers and family members, in addition to persons with MS. The findings have several implications. First, the study reiterates the value of MS relational networks, recognizing them as a valuable resource that needs to be resourced and considered in policy development, especially when preparing for future crises and chronic community stressors. Second, ensuring such preparedness requires strong investment into carer‐centred interventions that bolster carer resilience and enhance their well‐being. Finally, this study recognizes the importance of coping and adaptive responding that can build resilience, which service providers may miss if focusing extensively on challenges. To this end, service providers could more actively adopt a strength‐based approach, recognizing and supporting these strategies when engaging with persons with MS and their carers. Investing in these changes will benefit both carers and persons with MS as community stressors unfold.

## CONFLICT OF INTEREST

The authors declare no conflict of interest.

## ETHICS STATEMENT

The study was approved by the Murdoch University Human Research Ethics Committee (Approval 2019/057). Participants provided informed consent before participation and provided verbal consent at the beginning of each interview.

## Data Availability

The data set from the current study is not openly accessible as participant consent was given with the understanding that access to the data was only made available to the research team. Requests regarding the data set can be sent to helen.correia@murdoch.edu.au.

## References

[1] Milazzo A , Giles L , Parent N , McCarthy S , Laurence C . The impact of non‐pharmaceutical interventions on COVID‐19 cases in South Australia and Victoria. Aust N Z J Public Health. 2022;46(4):482‐487. 10.1111/1753-6405.13249 35557482 PMC9348509

[2] Manacorda T , Bandiera P , Terzuoli F , et al. Impact of the COVID‐19 pandemic on persons with multiple sclerosis: early findings from a survey on disruptions in care and self‐reported outcomes. J Health Serv Res Policy. 2021;26(3):189‐197. 10.1177/1355819620975069 33337256 PMC8182334

[3] Campbell JA , Simpson S , Ahmad H , Taylor BV , van der Mei I , Palmer AJ . Change in multiple sclerosis prevalence over time in Australia 2010–2017 utilising disease‐modifying therapy prescription data. Mult Scler J. 2020;26(11):1315‐1328. 10.1177/1352458519861270 31347952

[4] McGinley MP , Gales S , Rowles W , et al. Expanded access to multiple sclerosis teleneurology care following the COVID‐19 pandemic. Mult Scler J Exp Transl Clin. 2021;7(1):205521732199746. 10.1177/2055217321997467 PMC7934057 33738110

[5] Colais P , Cascini S , Balducci M , et al. Impact of the COVID‐19 pandemic on access to healthcare services amongst patients with multiple sclerosis in the Lazio region, Italy. Eur J Neurol. 2021;28(10):3403‐3410. 10.1111/ene.14879 33896086 PMC8250799

[6] Parkinson A , Drew J , Hall Dykgraaf S , et al. ‘They're getting a taste of our world’: a qualitative study of people with multiple sclerosis' experiences of accessing health care during the COVID‐19 pandemic in the Australian Capital Territory. Health Expect. 2021;24(5):1607‐1617. 10.1111/hex.13284 34227728 PMC8483188

[7] Learmonth YC , Hunter A , Gibbs L , Walker D , Kermode AG , Marck CH . The impact of the Australian Black Summer Bushfires and the COVID‐19 pandemic on wellbeing in persons with multiple sclerosis; preparation for future and ongoing crises. Disabil Rehabil. 2022:1‐14. 10.1080/09638288.2022.2037756 35166613

[8] Marck C , Hunter A , Heritage B , et al. The effect of the Australian bushfires and the COVID‐19 pandemic on health behaviours in people with multiple sclerosis. Mult Scler Relat Disord. 2021;53:103042. 10.1016/j.msard.2021.103042 34091177 PMC8451990

[9] Kalron A , Dolev M , Greenberg‐Abrahami M , et al. Physical activity behavior in people with multiple sclerosis during the COVID‐19 pandemic in Israel: results of an online survey. Mult Scler Relat Disord. 2021;47:102603. 10.1016/j.msard.2020.102603 33246261 PMC7588318

[10] Alschuler KN , Roberts MK , Herring TE , Ehde DM . Distress and risk perception in people living with multiple sclerosis during the early phase of the COVID‐19 pandemic. Mult Scler Relat Disord. 2021;47:102618. 10.1016/j.msard.2020.102618 33186805 PMC7644263

[11] Ramezani N , Ashtari F , Bastami EA , et al. Fear and anxiety in patients with multiple sclerosis during COVID‐19 pandemic; report of an Iranian population. Mult Scler Relat Disord. 2021;50:102798. 10.1016/j.msard.2021.102798 33571791 PMC7982777

[12] Talaat F , Ramadan I , Aly S , Hamdy E . Are multiple sclerosis patients and their caregivers more anxious and more committed to following the basic preventive measures during the COVID‐19 pandemic? Mult Scler Relat Disord. 2020;46:102580. 10.1016/j.msard.2020.102580 33296977 PMC7550868

[13] Bonavita S , Sparaco M , Russo A , Borriello G , Lavorgna L . Perceived stress and social support in a large population of people with multiple sclerosis recruited online through the COVID‐19 pandemic. Eur J Neurol. 2021;28(10):3396‐3402. 10.1111/ene.14697 33368849

[14] Motolese F , Rossi M , Albergo G , et al. The psychological impact of COVID‐19 pandemic on people with multiple sclerosis. Front Neurol. 2020;11:580507. 10.3389/fneur.2020.580507 33193033 PMC7662111

[15] Zarghami A , Hussain MA , Campbell JA , et al. Psychological impacts of COVID‐19 pandemic on individuals living with multiple sclerosis: a rapid systematic review. Mult Scler Relat Disord. 2022;59:103562. 10.1016/j.msard.2022.103562 35149393 PMC8786442

[16] Giménez‐Llort L , Martín‐González JJ , Maurel S . Secondary impacts of COVID‐19 pandemic in fatigue, self‐compassion, physical and mental health of people with multiple sclerosis and caregivers: the Teruel study. Brain Sci. 2021;11(9):1233. 10.3390/brainsci11091233 34573254 PMC8467200

[17] Reguera‐García MM , Liébana‐Presa C , Álvarez‐Barrio L , Alves Gomes L , Fernández‐Martínez E . Physical activity, resilience, sense of coherence and coping in people with multiple sclerosis in the situation derived from COVID‐19. Int J Environ Res Public Health. 2020;17(21):8202. 10.3390/ijerph17218202 33172022 PMC7664264

[18] Carotenuto A , Scandurra C , Costabile T , et al. Physical exercise moderates the effects of disability on depression in people with multiple sclerosis during the COVID‐19 outbreak. J Clin Med. 2021;10(6):1234. 10.3390/jcm10061234 33809698 PMC8002261

[19] Parkinson A , Brunoro C , Leayr J , et al. Intertwined like a double helix: a meta‐synthesis of the qualitative literature examining the experiences of living with someone with multiple sclerosis. Health Expect. 2022;25(3):803‐822. 10.1111/hex.13432 35118764 PMC9122458

[20] Cohen SA , Kunicki ZJ , Drohan MM , Greaney ML . Exploring changes in caregiver burden and caregiving intensity due to COVID‐19. Gerontol Geriatr Med. 2021;7:233372142199927. 10.1177/2333721421999279 PMC7919204 33718523

[21] Beach SR , Schulz R , Donovan H , Rosland AM . Family caregiving during the COVID‐19 pandemic. Gerontologist. 2021;61(5):650‐660. 10.1093/geront/gnab049 33847355 PMC8083337

[22] Muldrew DHL , Fee A , Coates V . Impact of the COVID‐19 pandemic on family carers in the community: a scoping review. Health Soc Care Community. 2021;30:1275‐1285. 10.1111/hsc.13677 34888980

[23] Ortelli P , Ferrazzoli D , Versace V , Saltuari L , Sebastianelli L . The need for psychological, caregiver‐centered intervention in the time of COVID‐19. Alzheimers Dement (N Y). 2021;7(1):12166. 10.1002/trc2.12166 PMC8114983 34013019

[24] Learmonth YC , Motl RW , Sandroff BM , Pula JH , Cadavid D . Validation of patient determined disease steps (PDDS) scale scores in persons with multiple sclerosis. BMC Neurol. 2013;13(1):37. 10.1186/1471-2377-13-37 23617555 PMC3651716

[25] Pakenham KI . The nature of caregiving in multiple sclerosis: development of the caregiving tasks in multiple sclerosis scale. Mult Scler J. 2007;13(7):929‐938. 10.1177/1352458507076973 17881402

[26] Braun V , Clarke V . Using thematic analysis in psychology. Qual Res Psychol. 2006;3(2):77‐101. 10.1191/1478088706qp063oa

[27] Nowell LS , Norris JM , White DE , Moules NJ . Thematic analysis: striving to meet the trustworthiness criteria. Int J Qual Methods. 2017;16(1):160940691773384. 10.1177/1609406917733847

[28] Byrne D . A worked example of Braun and Clarke's approach to reflexive thematic analysis. Qual Quant. 2021;56:1391‐1412. 10.1007/s11135-021-01182-y

[29] Tong A , Sainsbury P , Craig J . Consolidated criteria for reporting qualitative research (COREQ): a 32‐item checklist for interviews and focus groups. Int J Qual Health Care. 2007;19(6):349‐357. 10.1093/intqhc/mzm042 17872937

[30] Morrison EH , Michtich K , Hersh CM . How the COVID‐19 pandemic has changed multiple sclerosis clinical practice: results of a nationwide provider survey. Mult Scler Relat Disord. 2021;51:102913. 10.1016/j.msard.2021.102913 33839482 PMC7969827

[31] Mazzucchelli TG , Kane RT , Rees CS . Behavioral activation interventions for well‐being: a meta‐analysis. J Posit Psychol. 2010;5(2):105‐121. 10.1080/17439760903569154 20539837 PMC2882847

[32] Portocarrero FF , Gonzalez K , Ekema‐Agbaw M . A meta‐analytic review of the relationship between dispositional gratitude and well‐being. Pers Individ Dif. 2020;164:110101. 10.1016/j.paid.2020.110101

